# Economic burden of family caregiving for elderly population in southern Ghana: the case of a peri-urban district

**DOI:** 10.1186/s12939-016-0511-9

**Published:** 2017-01-14

**Authors:** Stephen Tettey Nortey, Genevieve Cecilia Aryeetey, Moses Aikins, Djesika Amendah, Justice Nonvignon

**Affiliations:** 1 Sinel Specialist Hospital, Tema, Ghana; 2 Department of Health Policy, Planning and Management, School of Public Health, College of Health Sciences, University of Ghana, Legon, Ghana; 3 African Population and Health Research Centre, Nairobi, Kenya

**Keywords:** Family caregivers, Economic burden, Elderly population, Health systems, Ghana

## Abstract

**Background:**

Health systems in low and lower-middle income countries, particularly in sub-Sahara Africa, often lack the specialized personnel and infrastructure to provide comprehensive care for elderly/ageing populations. Close-to-client community-based approaches are a low-cost way of providing basic care and social support for elderly populations in such resource-constrained settings and family caregivers play a crucial role in that regard. However, family caregiving duties are often unremunerated and their care-related economic burden is often overlooked though this knowledge is important in designing or scaling up effective interventions. The objective of this study, therefore, was to estimate the economic burden of family caregiving for the elderly in southern Ghana.

**Methods:**

The study was a retrospective cross-sectional cost-of-care study conducted in 2015 among family caregivers for elderly registered for a support group in a peri-urban district in southern Ghana. A simple random sample of 98 respondents representative of the support group members completed an interviewer-administered questionnaire. Costs were assessed over a 1-month period. Direct costs of caregiving (including out-of-pocket costs incurred on health care) as well as productivity losses (i.e. indirect cost) to caregivers were analysed. Intangible costs were assessed using the 12-item Zarit burden interview (ZBI) tool and the financial cost dimension of the cost of care index.

**Results:**

The estimated average cost of caregiving per month was US$186.18, 66% of which was direct cost. About 78% of the family caregivers in the study reported a high level of caregiving burden (as measured with the ZBI) with females reporting a relatively higher level than males. Further, about 87% of the family caregivers reported a high level of financial stress as a result of caregiving for their elderly relative.

**Conclusion:**

The study shows that support/caregiving for elderly populations imposes economic burden on families, potentially influencing the economic position of families with attendant implications for equity and future family support for such vulnerable populations.

## Background

The proportion of elderly (also referred to as ageing) populations is increasing globally; it is estimated that by 2050, around two billion persons in the world will be aged 60 years and over, with 400 million aged 80 years and over [1]. Of this population, 80% will live in low and middle income countries (LMICs). In Europe, the proportion of elderly people is estimated to reach 33.6% by 2050, while in sub-Saharan Africa the projected proportion is 8.3% [2]. The growth in the elderly population is faster in LMICs compared to higher income countries. In Ghana, for example, the absolute number of the elderly has increased seven-and-half times in the last five decades, from 213,477 in 1960 to 1,643,381 in 2010—representing 4.5 and 6.7% of the total population respectively [3]. The demographic trend and prediction of sharp rises in the numbers and proportion of the elderly has led to considerable interests in issues of population ageing. Such overwhelming interest has been driven, in part, by the notion that costs to society in general, and to families in particular, will escalate with the increasing population of dependent elderly people [2].

In many LMICs including Ghana, families have played a crucial role in caring for the elderly. This caregiving arrangement, however, is currently undergoing extensive changes due to several factors including: a weaker extended family system; a rural-urban within country migration and outmigration from Ghana, often leaving the elderly with limited support; general difficulties in global economic conditions with families (especially younger people) becoming busier and making little time for the elderly.

Studies have documented the psychological, social and health consequences that caregivers of elderly populations face [4–6] and have provided an impetus to the development of social policies to support the caregivers in some countries. Examples are caregiver support groups and respite services in many countries paid for or subsidized by the state [5]. But those studies have examined the economic burden of caring for elderly population in mostly high income countries [5, 7, 8], and usually focused on the economic effect on employers, national income and cost to society, with limited emphasis on economic burden to families and primary caregivers. This focus is partly because family caregiving and support to the elderly usually lie outside the market economy and is socially and politically invisible [9]. Hence, its economic value is not generally acknowledged. Consequently, little is known about the economic burden of family caregiving for the elderly in Ghana in particular and LMICs in general.

In 2010, a National Ageing Policy [10] for Ghana was adopted, seeking to achieve overall social, economic and cultural re-integration of elderly people into mainstream society, and to enable them to participate fully in the national development process. However, not much has happened in terms of implementation of the policy. One key strategy in achieving this goal is to strengthen the family support systems for the elderly. Hence, it is important to assess such burden so as to highlight it and appropriately direct economic and social interventions aimed at encouraging family support to caregivers. Thus, this study aimed at estimating the economic burden of family caregiving for the elderly in southern Ghana.

In this study, economic burden refers to any burden (resulting from caring for the elderly) that has implication on the economic welfare of individuals. Such burden includes direct costs (i.e. payments or expenses that caregivers made on health and related care), indirect costs or productivity losses that result from absent from normal productive work (which also normally affects the earning ability of those incurring this costs). Other burdens analysed in the study include intangible costs (i.e. costs that are difficult to quantify due to their nature).

## Methods

### Study design

A retrospective cross-sectional cost-of-care design was used.

### Study area

The study was conducted in the Ga-East Municipality in the Greater Accra Region of Ghana. The municipality has a population of 147,742 out of which 74,755 (51%) are females [3]. The elderly accounts for 4.5% (6650) of the population in the municipality.

### Study population, sample size and sampling

Family caregivers for the elderly living within the Ga-East Municipality of Accra between May, 2015 and June, 2015 formed the study population.

The area was considered for the study due to the presence of a support group for the aged which served as an entry into the vicinity. The family caregivers recruited for the study were caregivers of elderly persons recruited from the Akrowa Aged Life Foundation (AALF) register. The AALF is a not-for-profit organisation which undertakes home visits to support care of the elderly. The registry had a total number of 160 elderly persons living within the Ga-East Municipality (as at 2014). The study assumed that most of these elderly people had family caregivers. With this finite population (160), the sample size for the caregivers was determined by adopting the following statistical formula for minimum sample size calculation [11]:

$$ \mathrm{n}=\frac{\mathrm{N}}{1 + \mathrm{N}\ {\left(\mathrm{e}\right)}^2} $$

Where:

N = the sampling frame (i.e. the total number of elderly people on the AALF register)

e = the margin of error. 7% (0.07) was used.

n = the minimum sample size of caregivers needed for the study.

From the above:

$$ \mathrm{n} = \frac{160}{1 + 160\ (0.07)2} $$

Based on the above calculation, a minimum sample size of 89 was obtained from the target group. This was approximated to 90 and using a non-response rate of 20%, the sample size was further increased to 108 caregivers. The additional 18 was to make room for possible incomplete questionnaires and non-respondents, especially due to the possibility of not meeting some of the caregivers at home as at the time of visit. A total of 98 questionnaires were successfully completed, with a response rate of 91%.

Using the Foundation’s register as a sampling frame, a random sample of 108 identification numbers was drawn. Subsequently, using their residential addresses and contact information and with the aid of staff of the AALF, caregivers of the sampled elderly persons were contacted and interviewed upon consenting to the study. Caregivers who did not meet the inclusion criteria, were not located or declined to be interviewed were replaced with the next available caregiver on the list who qualifies for the study. For the purpose of this study, a family caregiver refers to a person who provided assistance, in the past 1 month, with at least one caregiving task because of a long-term health condition of the care recipient without receiving any financial payment. Caregivers receiving monetary payment for caregiving to the elderly, caregivers below the age of 18 years and caregivers who have spent less than a month providing care for the elderly were excluded from the study.

### Data collection and tools

A structured interviewer-administered questionnaire with both open-ended and closed-ended questions was used in collecting data for this study. The questionnaire included questions on: indirect costs to caregivers (family caregivers were asked to indicate the amount of time they spend on various caregiving activities within a typical week. The activities included personal care for the elderly, household activities, travel/transportation and time spent with the care recipient in keeping company); direct health care cost to caregivers (family caregivers were asked to indicate their out-of-pocket expenses in the areas of medical care, household supplies, residential care, transportation/travel and financial transfer within the past 1 month); intangible cost. Intangible cost was assessed using the 12-item Zarit Burden Interview (ZBI) tool [12] and the financial dimension of the cost of care index (CCI) questionnaire [13]. The 12-item ZBI covers areas on personal strain and role strain. The caregivers were asked to indicate the impact of the care receiver’s condition on his or her life by specifying how often they felt the way that was described by each item. Each of the items had a 5-point scale with 0 = never, 1 = rarely, 2 = sometimes, 3 = quite frequently, and 4 = always. The financial dimension of the cost of care index covers areas on financial stress to the family caregiver. Family caregivers were asked to respond to each question by choosing an answer on a 4-point scale- strongly disagree, disagree, agree and strongly agree, with corresponding scores of 1 to 4 respectively. Table 1 provides details of the study variables.

**Table 1:** Description of study variables

Cost type	Cost categories	Description
Direct cost	Medical cost	
	Medical care	Cost associated with medications, health supplies, consultation, treatments and therapies.
	Non-medical cost	
	Residential care	Cost associated with rent, utilities and other housing expenses
	Household supplies	Cost associated with food, clothing, toiletries and personal items.
	Travel/transportation	Cost associated with travelling to, with or for the care recipient.
	Financial transfer	Cost associated with occasional monetary transfer from caregiver to care recipient.
Indirect cost	Productivity loss as a result of;	
	Time spent on personal care	Time spent helping care recipient with feeding, going to the toilet, bathing, changing bandages and giving medicines.
	Time spent on household activities	Time spent on meal preparation, washing, cleaning or shopping on behalf of care recipient.
	Time on travel/transportation	Time spent in travelling to, with or for the care recipient.
	Time spent with care recipient	Time spent being a companion; facilitating social interactions and reducing social isolation.
Intangible cost	Caregiving burden	
	Caregiving burden	Zarit burden interview score
	Financial stress	Financial dimension of cost of care index score

Data collection was carried out by the lead author assisted by trained research assistants. The questionnaires were administered to family caregivers individually in the form of a face-to-face interview.

### Data analysis

Costs (economic burden) were analysed from the caregiver perspective and for a 1 month period (i.e. preceding the data collection). Costs were categorized into direct cost, indirect cost and intangible. Direct costs were further categorised into medical cost and non-medical cost (Table 1).

Indirect cost constitutes productivity losses due to lost work time and was estimated using the human capital approach which measures output losses by lost earnings. The total indirect cost per month by family caregivers was derived by multiplying the total hours spent by the national daily minimum wage of GHS7.04 (or GHS0.88 per hour) for family caregivers who were employed in the formal sector and the local daily casual labour wage of GHS15.04 (or GHS1.88 per hour) for family caregivers in the informal sector. Indirect costs incurred by students/apprentices and caregivers who were unemployed are presented in terms of lost hours but not valued.

The total cost of family caregiving for the elderly per month was estimated by summing total direct costs per month and total indirect costs per month. Average cost was derived by dividing total costs by the number of respondents. All estimated costs were further converted into 2015 US$ using an exchange rate of GHS4.16 (the exchange rate at the time this study was conducted).

With respect to intangible cost, ZBI has a range of total scores from 0 to 48 with higher scores representing a higher level of caregiving burden. Bedard et al. [12] proposed a cut of point of 16 to distinguish low burden (i.e. <16) and high burden (i.e. 16 and above). The financial dimension of the CCI has a total score range of 4 to 16, with higher scores indicating higher level of financial stress. Proportions of family caregivers who reported positively on each of the four items were estimated and reported.

The data were analysed using Microsoft Excel 2010 and STATA 12. Descriptive statistics were used to present background characteristics of participants and to describe the intangible cost of caregiving.

## Results

### Background characteristics of caregivers and care recipients

Table 2 shows that females constituted 68% of caregivers, and 43% of caregivers were in age group 30 and 44 years. About 8% of caregivers were elderly themselves (i.e. above 60 years). Further, about 56% of caregivers were self-employed and about 25% were either unemployed, students or apprentices. Table 2 further shows that about 94% of respondents reported personal monthly income of less than US$240.00. About 85% of the respondents identified themselves as primary caregivers whereas the remaining respondents considered themselves as secondary caregivers. Most the caregivers were children (55%) or grandchildren (24%) of the care recipients. The spousal caregivers accounted for 6% of all the respondents. About 79% of the caregivers co-resided with their care recipient and 13% reported living nearby. Approximately 62% of the caregivers indicated that their finances have gotten worse as a result of caregiving for their elderly relative.

**Table 2:** Caregivers’ background characteristics

Background characteristic	Frequency (n)	Percent (%)
Sex		
Male	31	31.6
Female	67	68.4
Age		
18–29	27	27.6
30–44	42	42.9
45–60	21	21.4
Above 60	8	8.1
Marital Status		
Single	35	35.7
Married	45	45.9
Divorced	10	10.2
Widowed	8	8.2
Education		
No education	6	6.1
Primary	5	5.1
Middle/JHS/JSS	60	61.2
SSS/SHS	12	12.3
Tertiary	15	15.3
Employment		
Self employed	55	56.1
Private sector	13	13.3
Public sector	6	6.1
Unemployed	15	15.3
Student/Apprentice	9	9.2
Relationship with CR		
Spouse	6	6.1
Child	54	55.1
Grandchild	23	23.5
Sibling	14	14.3
Daughter/Son-in-law	1	1
Caregiving status		
Primary CG	83	84.7
Secondary CG	15	15.3
Duration of care		
Less than 1 year	8	8.2
1–4 years	56	57.2
5–9 years	22	22.4
10 years and above	12	12.2
Residence status		
Long distance CG	8	8.2
Co-resident CG	77	78.5
Nearby CG	13	13.3
CG personal income (US$)		
0–24	9	9.2
25–119	67	68.4
120–240	16	16.3
241–480	4	4.1
480 and above	2	2
Impact on CG finances		
Gotten worse	61	62.2
Stayed the same	36	36.7
Gotten better	1	1.1

Two-thirds (67%) of the elderly care recipients were females (Table 3) and their age ranged from 60 to 106 years respectively, with median age of 78 years. About 28% of care recipients were above 85 years old. The proportion of care recipients who required assistance with at least one form of activity for daily living (ADL) was 52%. All the care recipients in the sample required assistance with some form of instrumental activity for day living (IADL).

**Table 3:** Background characteristics of care recipients

Background characteristic	Frequency (n)	Percent (%)
Sex		
Male	32	32.6
Female	66	67.4
Age		
60–74 (young old)	36	36.7
75–84 (old-old)	35	35.7
85 and above (very old)	27	27.6
Assistance with		
ADL	51	52.0
IADL	98	100

*IADL* Instrumental activity for day living, *ADL* Activity for daily living

### Cost of caregiving

The average caregiving time per month for a caregiver was estimated at 219.5 (±25.9) hours (Table 4). Of that time, most of it was spent on personal care. All family caregivers in the sample spent a total of 17,900 h on caregiving for their care recipients within the month.

**Table 4:** Time spent on caregiving

Time	Frequency n (%)	Average time spent per month mean h (SD)	Total time spent per month (h)—all caregivers
Time spent on personal care	97 (99)	101.3 (9.1)	9796
Time spent on household activities	83 (83)	69.4 (8.7)	5760
Time spent on travel/transportation	34 (35)	22.0 (3.8)	748
Time spent with care recipient	62 (63)	26.8 (4.3)	1596
TOTAL		**219.5 (25.9)**	**17,900**

The average monthly cost of family caregiving for the elderly was US$186.18. The total monthly cost of caregiving for the elderly in the study sample was estimated as S$14,568.03. For average and total costs, direct cost constituted about 66% and indirect cost about 34% (Table 5).

**Table 5:** Cost of caregiving for the elderly

Cost category	Frequency (n)	Total Amount (US$)	Average Amount (US$)	Cost Profile (%)
DIRECT COST				
Medical				
Medical care	78	2068.27	26.51	14.2
Non- medical				
Household supplies	97	6021.63	62.07	41.3
Residential care	83	743.03	8.92	5.1
Transport	44	527.4	11.2	3.6
Financial transfer	40	280.05	7	1.9
Total direct cost		**9640.38**	**115.72**	**66.2**
INDIRECT COST				
Valued time spent on personal care	73	2722.04	28.06	18.7
Valued time spent on household activities	64	1563.85	24.43	10.7
Valued time spent on travel/transportation	24	190.46	7.94	1.3
Valued time spent with care recipient	45	451.31	10.03	3.1
Total indirect cost		**4927.65**	**70.46**	**33.8**
TOTAL		**14,568.03**	**186.18**	**100**

About 77.6% of the family caregivers reported a high burden (on the ZBI) with the remaining (22.4%) reporting a low burden. For both male and female, family caregivers reported a high caregiving burden but females reported a relatively higher burden level than males.

The results of the individual items on the financial dimension of the cost of care index indicated that 80% of family caregivers agreed that caregiving for the care recipient was causing them to dip into their own savings. Again, 66% of the family caregivers indicated that they and their families could not afford those “little extras” because of expenses to care for the care recipient. Over a half (59%) indicated that their family or they had to give up necessities because of the expense to provide care. More than three quarters (87%) of the caregivers indicated that caring for the care recipient was too expensive.

## Discussion

The findings of this study suggest that direct cost (out-of-pocket expenses) is a major source of cost of family caregivers of the elderly. The estimated average direct cost per month was US$115.72. However, with 94% of caregivers reporting monthly income of less than US$240, it is clear that at least 48% of monthly income is spent on caregiving for an elderly person in the household. This is indicative of a high financial stress and may consequently explain the high burden level reported by most caregivers in this study. Similarly, Duncan et al. [14] reported that those caregivers with less financial means have significant care-related expenses that represent a larger proportion of their household incomes compared to those caregivers with higher incomes.

Non-medical costs accounted for the larger amount of the direct cost, with cost of household supplies constituting the highest proportion of the direct cost. The estimated average monthly cost of household supplies to a family caregiver was US$62.07. This constituted 41.3% of the total cost and more than half of the estimated average monthly out-of-pocket expenses of most of the caregivers in this study. These findings suggest that household supplies account for a greater amount of the cost incurred in taking of an elderly relative. It is important to state, however, that it is possible that the burden of direct costs did not entirely fall on the primary caregiver, as other family members may have contributed to that expense. Thus, the direct costs likely represent a shared burden. However, there is little information about the extent to which these expenses may create financial and other hardships for these family caregivers. There is therefore the need for further investigation into this category of care-related cost to family caregivers.

Financial transfer constituted the smallest proportion (1.9%) of the total out-of-pocket cost of caregiving for the elderly in this study. There has not been sufficient research on financial transfer from caregiver to care recipient to enable comparison across studies. A study focusing on the elderly in the United States reported that 15% of family caregivers transferred an average of US$58 to their care recipients in the past month [15]. This current study estimated that 41% of the family caregivers spent an average amount of US$7.00 on financial transfer to their care recipients. This difference may be explained in part by the fact that three out of four (about 79%) of the family caregivers in this study co-resided with their care recipient. Hence, they were directly responsible for the purchase of any item the care recipient might require and may not need to give out money directly to the care recipient.

In general, this study adds credence to the already existing evidence that care-related out-of-pocket cost of family caregivers for the elderly is substantial. It constitutes the larger proportion of the total cost of caregiving and can threaten the economic security of most family caregivers, particularly those with low income earnings. Further investigations of both immediate and long term outcomes of out-of-pocket costs of family caregivers are needed to determine the extent to which the caregivers may be at high risk of poverty and inability to sustain their caregiving obligations.

With respect to productive time (hours) lost to caregiving, on average, the family caregivers in this study spent 219.5 (±25.9) hours in a month or 54.9 (±6.5) hour per week giving care to their elderly relative. In contrast, in a UK survey, family caregivers for the elderly provided about 20.0 h of care per week [16]. Another study in the US reported that family caregivers provided an average of 21.0 h of care per week [17]. This relatively high level of time spent in caregiving by family caregivers in this study can be explained in part by the fact that majority (79%) of them were also co-resident caregivers. It has been reported that co-resident caregivers particularly find it difficult to disaggregate time spent in care tasks from other household duties [7]. This may lead to over reporting of time spent by the family caregivers in this study in giving care to the elderly. Notwithstanding, given that the infrastructure in developing countries hardly supports independence on the part of the care recipient to undertake tasks, it is more likely that caregivers in this setting actually spend more time on care-related tasks compared to those in developed countries. These variations may be attributed to the differences in jurisdiction, the inconsistencies in the operational definition of caregiving labour (e.g., which caregiving tasks are included) and the differences in data collection methodology with most of these previous studies being telephone surveys. However, a better comparison would have been with studies conducted in sub-Saharan Africa with relatively similar background characteristics of family caregivers. However, there has been much less investigation into the economic burden of family caregiving for the elderly in Africa reflecting an ongoing reluctance to make public the ‘private’ work of families [18]. Further investigation into this may be needful in other African countries to enable comparison across similar jurisdiction.

The time spent in care for the elderly results in reductions in time spent in other activities such as employment, education, accumulation of human capital and personal care or leisure. Empirically, studies have shown negative effects of family caregiving responsibilities on hours worked and participation in the labour market [19, 20]. Studies have also shown that family caregivers with substantial care responsibilities are less likely to be employed than non-caregivers with lighter responsibilities [21, 22], and they are also more likely to work fewer hours and experience wage penalties [20, 22]. These previous findings are consistent with the findings of this current study. Majority (56.1%) of the family caregivers in this study were employed in the informal sector. Similarly, majority (94%) of the family caregivers in this study earned less than US$240 per month. What is not clear, however, is whether, due to their caregiving responsibilities, family caregivers reduce their hours for work and participation in the labour market, consequently leading to a reduction in their monthly income.

Estimates of the monthly time spent in family caregiving to the elderly in this study suggest that many family caregivers undertake the equivalent of a part-time or even a full time job to give care to their elderly relative. Aggregation of this time spent in family caregiving for the elderly in any national economy can be equivalent of several full-time jobs. Hence, the time costs of care should be a major focus in economic evaluations, especially in subsequent studies, in an attempt to account for the national cost of family caregiving in Ghana. This current study therefore estimated the average indirect cost of family caregiving for the elderly per month at US$70.46. This constitutes one-third of the total cost of caregiving for the elderly in this study. Indeed, this represents a substantial amount of foregone benefits or earnings to the family caregivers in this study. Few attempts have been made to estimate the value of unpaid care. Among them, Feinberg et al. valued time of family caregivers at US$450 billion per year in Canada [5] in the year 2011. Similarly, Hollander et al. [23] also estimated costs of care labour between US$9 million and US$21 million in Canada. However, it should be noted that these studies were national surveys with a very large sample of caregivers and these estimates were calculated per year. Unlike this current study with a relatively small sample of caregivers and with estimates of indirect cost for only a month of care to the elderly.

About 15% of respondents in this study were unemployed. However, a question that emerges from this study is the direction of causation between caregiving for the elderly and participation in the paid labour force. Do people leave paid employment to assume caregiving (employment status as an outcome), or do they take on caregiving in the absence of employment opportunities or employment (employment status as a determinant)? Further investigation is needed to clarify the complex relationship between caregiving and employment opportunities or employment.

With respect to the ZBI, 78% of the family caregivers in this study reported a high level of burden. This finding echoes the findings in some previous studies which indicated that family caregivers are more likely to have a higher burden when compared to those without caring responsibilities [24]. This is particularly the case for family caregivers providing intensive levels of care [25]. Considering the fact that more than half (52%) of the elderly in this study needed assistance with one form of ADL or the other, it was not surprising that most of the caregivers in this study reported a high level of caregiving burden.

Additionally, this study also showed that caregiving burden was relatively higher among females than males. This compares with the findings of some previous studies that shows that the traditional role of caregiving is often expected of and performed by females [26, 27]. Other explanations of the higher levels of caregiving burden experienced by female caregivers probably include as indicated in previous studies, the multiple caring roles of women [28] and other gender-related challenges, such as spending more time with care recipient than male caregivers [29], receiving less assistance with caregiving tasks, and spending more time on intensive personal care and domestic chores [30]. All these explanations point to the need for support for family caregivers especially female caregivers. Society tends to feel very comfortable with treating family caregiving particularly care provided by females as a familial obligation [6], without paying adequate attention to the needs of female caregivers. It is important for policy makers to address the unique caregiving stress and challenges family caregivers particularly females face while providing care.

Family caregivers in this study also indicated a high level of financial stress. Indeed, nearly two-thirds (62%) of the family caregivers in this study reported a worse state of financial well-being as a result of caregiving for their elderly relative. Majority of the family caregivers in this study reported dipping into saving (80%), not being able to afford those little extras (66%), and giving up necessities (59%). These findings compares slightly with the findings of Lai [6] who reported proportions of 40, 40 and 38.5% respectively. This high incidence of financial stress among these family caregivers was however, expected. Previous studies have consistently shown that, among family caregivers, caring for 20 h or more per week was associated with a higher risk of financial stress [22]. Most of the caregivers in this study were primary caregivers who provided more than 20 h of care per week. This could reduce participation in the labour market leading to a reduction in their monthly income and consequently increased financial stress. It was therefore not surprising that an overwhelming majority (87%) of the family caregivers in this study indicated that caring for their elderly relative was too expensive.

In general, the evidence of high burden and increased financial stress for family caregivers in this study is compelling and it is likely to be an indicator of substantial costs associated with caregiving for the elderly. Further studies are required to identify the determinants and outcomes of this burden and financial stress that caregivers encounter and how policy could assist in defraying the expenses associated with them.

Some limitations of the study are worth mentioning. Although a random sample was used, the localization of the sample makes it difficult to generalise findings to family caregivers in other localities in Ghana. Also, the study used a subjective method which predominantly depended on the recall abilities of the family caregivers and hence could have been affected by recall bias. Additionally, it is unclear whether the care recipients had accumulated assets that were sold or engaged in productive activities and the proceeds used for their care or that would “reimburse” their caregivers after the original owner passes on. Finally, it is unclear from the study whether the caregiving patterns change depending on the health of the receivers. These limitations could serve as a basis for further studies.

Future studies could explore the immediate and long term outcomes of the economic burden and financial stress as highlighted in this study on the caregivers and the elderly. These studies could investigate the extent to which family caregiving links with at the risk of current or future poverty and inability to sustain their caregiving obligations.

Despite those limitations, the study offers useful insights into the care of older Ghanaians that could be useful for policy

## Conclusions

The study shows that support/caregiving for elderly populations imposes economic burden on families, potentially influencing the economic position of families with attendant implications for future family support for such vulnerable populations.

The findings of the study have implications for social and developmental policies in Ghana. First, social protection programmes like the Livelihood Empowerment Against Poverty (LEAP), could be expanded to cover not only more elderly people but also family caregivers of these vulnerable people, especially those with high economic and caregiving burden Second, the overall social infrastructure and the health system need to be make provision for the elderly. The Ghanaian health care system in particular does not seem to highlight the special attention for geriatric populations; the public health care system reports no geriatric specialists and no institution to train such specialists. The social welfare system is recently gaining some attention with some social protection programmes targeting a portion of the elderly population (e.g. the National Health Insurance Scheme, LEAP). However, a holistic approach needs to be adopted and such approach could in the long run lessen the burden on families and encourage re-integration of elderly persons. Policy makers could consider tax incentives to the private sector to encourage them take on more corporate social responsibilities that specifically target elderly and other vulnerable populations.

The findings of this study also have implications for equity in health in the Ghanaian context. While efforts have been made to improve the delivery of health care services to elderly populations, in addition to providing other social support to the elderly, the burden that health care for the elderly places on the welfare of caregivers has seen little attention. Further, the study reported higher (intangible) burden on female compared to male caregivers, which raises questions on equity. Further investigations are needed on this aspect of caring for elderly populations.

## Abbreviations

AALF: Akrowa Aged Life Foundation
ADL: Activity for daily living
CCI: Cost of care index
IADL: Instrumental activity for day living
LEAP: Livelihood Empowerment Against Poverty
LMICs: Low and middle income countries
SSA: Sub-Saharan Africa
WHO: World Health Organization
ZBI: Zarit burden interview
